# Protective effect of L-pipecolic acid on constipation in C57BL/6 mice based on gut microbiome and serum metabolomic

**DOI:** 10.1186/s12866-023-02880-3

**Published:** 2023-05-20

**Authors:** Huan Li, Hong-yun Xiao, Li-ping Yuan, Bo Yan, Ying Pan, Ping-ping Tian, Wei-jie Zhang

**Affiliations:** 1 Department of Pediatrics, First Affiliated Hospital of Anhui Medical University, Hefei, 230022 Anhui China; 2 Fuyang Hospital of Anhui Medical University, Fuyang, 236000 Anhui China; 3 Department of Technology, Anhui Medical College, Hefei, 230022 Anhui China

**Keywords:** Functional constipation, Gut microbiota, Metabolite profiles, L-pipecolic acid, Children

## Abstract

**Background:**

Functional constipation (FC) in children affects their growth, development and quality of life. L-pipecolic acid (L-PA) was decreased in FC children based on gut microbiome and serum metabolomic. In this study, loperamide-induced constipation in mice was used to evaluate the effects of L-PA on constipated mice.

**Method:**

26 FC and 28 healthy children were recruited. Stool samples and serum samples were subjected to 16S rDNA sequencing and ultra-performance liquid chromatography/quadrupole time of flight (UPLC-Q/TOF-MS) approach, respectively. A loperamide-induced mouse constipation model was developed, and all mice were randomly divided into control (Con), loperamide (Lop) and L-PA (Lop + L-PA) treatment groups (6 mice per group). The mice in the Lop + L-PA group were given L-PA (250 mg/kg, once a day) and loperamide; the Lop group was given loperamide for 1 week, and the Con group was given saline. The fecal parameters and intestinal motility of mice in each group were detected. serum 5-HT levels and colon 5-HT expression were detected by ELISA and immunohistochemistry, respectively; qRT-PCR was used to detect the expression of AQP3 and 5-HT4R mRNA in each group.

**Results:**

45 differential metabolites and 18 significantly different microbiota were found in FC children. The α and β diversity of gut microbiota in FC children was significantly reduced. Importantly, serum L-PA was significantly reduced in FC children. The KEGG pathway enrichment were mainly enriched in fatty acid biosynthesis, lysine degradation, and choline metabolism. L-PA was negatively associated with *Ochrobactrum*, and N6, N6, N6-trimethyl-l-lysine was positively associated with *Phascolarcrobacterium*. In addition, L-PA improved the fecal water content, intestinal transit rate, and increased the serum 5-HT levels in constipated mice. Moreover, L-PA increased the expression of 5-HT4R, reduced AQP3, and regulated constipation-associated genes.

**Conclusions:**

Gut microbiota and serum metabolites were significantly altered in children with FC. The abundance of *Phascolarctobacterium* and *Ochrobactrum* and serum L-PA content were decreased in FC children. L-PA was found to alleviate the fecal water content, increase intestinal transit rate and the first black stool defecation time. L-PA improved constipation by increasing 5-HT and 5-HT4R expression while down-regulating AQP3 expression.

**Supplementary Information:**

The online version contains supplementary material available at 10.1186/s12866-023-02880-3.

## Background

Functional constipation (FC) is a gastrointestinal disorder in childhood with 29.6% worldwide prevalence [1]. The majority of children with constipation had no underlying physical disorders but in one-third of them problems continue after adolescence [2, 3]. FC in children is characterized by infrequent, hard, large, and painful bowel movements, and fecal incontinence [4]. Pain, fever, dehydration, inappropriate diet and fluid intake, psychological issues, toilet training, certain medicines, and a family history of constipation can all contribute to FC [5–7]. Treating children with FC remains a serious problem, due to the complexity of the pathogenesis.

Gut microbiota is a complex and dynamic biome in the human body that plays an important role in the host’s physiological functions [8]. Studies have reported significant variation in the intestinal and mucosa-associated microbiota in children with a history of constipation. A Japanese study showed that the diversity of mucosal microbiome in children with FC was structurally different from the healthy controls, and the mucosa-associated microbiome of FC was characterized by higher levels of *Bacteroidetes* (*Alistipes*)[9]. Tim et al. have found that the *Bacteroides fragilis*, *Bacteroides ovatus*, *Bifidobacterium longum*, *Parabacteroides species* were increased in patients with FC [10]. Microbial-based interventions, such as probiotics, antibiotics, fecal transplantation, and gut microbiota, have shown therapeutic potential towards constipation in animal models and clinically [11, 12]. An animal study showed that human fecal microbiota transplanted mice had higher colonic contractility and significantly shortened gastrointestinal transit time compared to germ-free mice [13]. Moreover, it has been proved that gut microbiota can affect the secretion and absorption of metabolites to promote the development of FC, such as bile acids, short-chain fatty acids and methane. Disruption of the microbiota usually causes metabolic changes that affect the physiological mechanisms of the host. The present study found that the abundance of *Phascolarctobacterium* and *Ochrobactrum* were decreased, and the serum L-pipecolic acid (L-PA) content was decreased in FC children.

L-PA is an important rigid cyclic non-protein amino acid. The content of L-PA was downregulated in patients with acute ischemic stroke [14]. L-PA was significantly reduced in the serum of ulcerative colitis patients, which reduced depressive-like behaviors in mice with colitis and alleviated the inflammatory cytokine levels in the colon, blood, and brain [15].

This study was designed to explain the pathogenesis of FC using 16S rDNA sequencing and UPLC-Q/TOF-MS approach, and to identify the key microbiota and metabolites in children with FC. A loperamide-induced mouse constipation model was developed by giving loperamide to mice, and the effect of L-PA on improving constipation was analyzed. Importantly, this is the first study to investigate the protective effect of L-PA on the constipation induced by loperamide in mice. This study lays a theoretical foundation for new drugs to prevent or treat FC.

## Results

### 16S rDNA sequence analysis

**Fig. 1:**
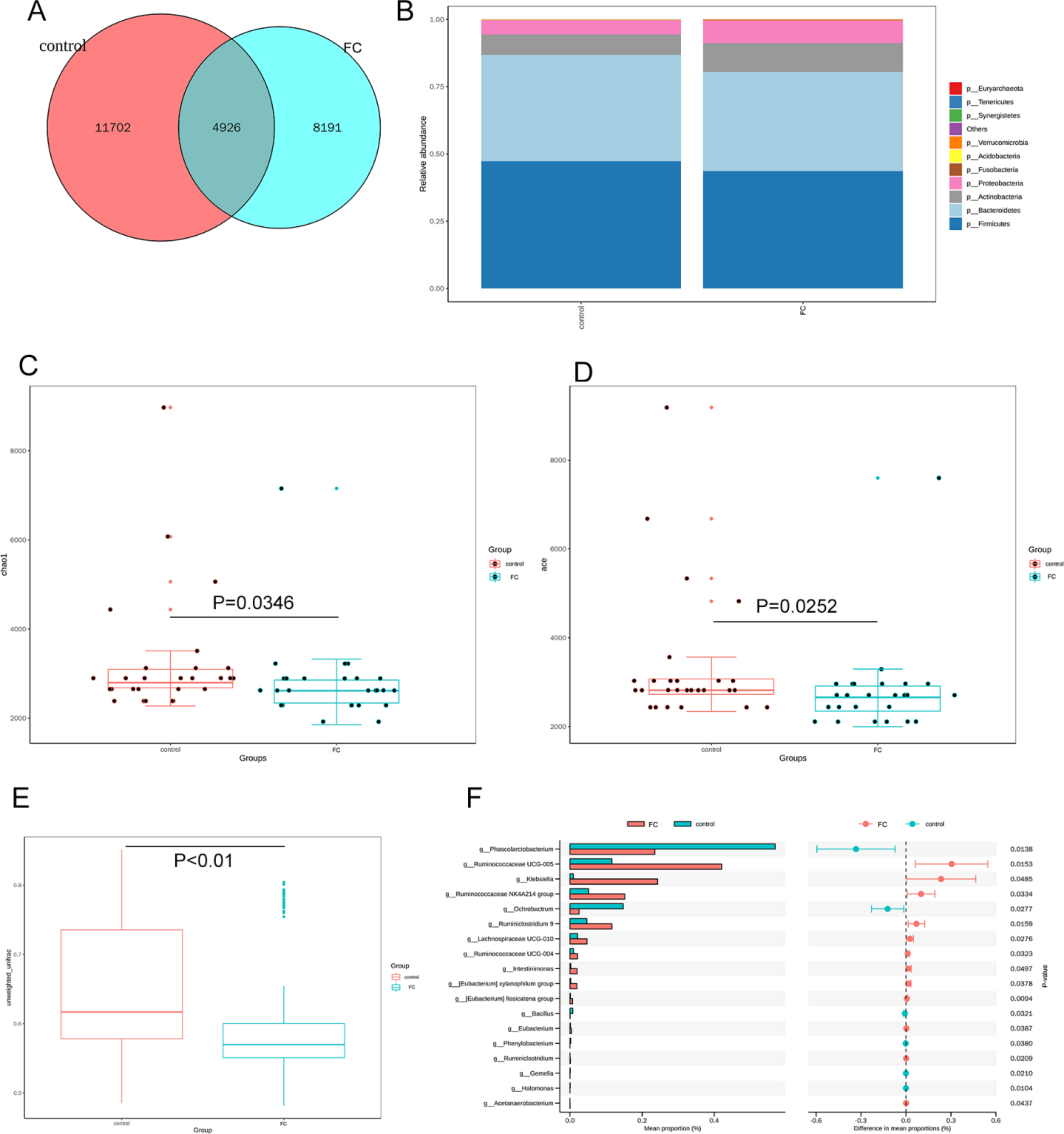
Alteration of gut microbiome. The common and unique OTUs of gut flora were shown in a Venn map between healthy controls and children with FC **(A)**. The abundance of species at the phylum level **(B)**. A chao1 **(C)** metric and an ace metric **(D)** were used to assess the α diversity. β diversity index was significantly lower in the functional constipation group compared with the control group **(E)**. The altered microbiome was processed by STAMP analysis **(F)**

According to the finding of the operational taxonomic units (OTUs) cluster analysis, a total of 13,117 OTUs were obtained in children with FC. There were 4926 common OTUs and 8191 unique OTUs in FC children (Fig. 1A). Distinct species were primarily clustered in six orders with ten genera (**Supplemental Fig. 1).** The results from the top 10 taxa at the phylum levels showed that the relative abundance of *Actinobacteria* and *Proteobacteria* increased while that of *Firmicutes* and *Bacteroidetes* decreased in the FC group (Fig. 1B). The α diversity was assessed using α diversity indices, including chao1and ace which showed a significant difference between the two groups (Fig. 1C.**D).** The α diversity of the gut microbiota was significantly lower in the children with FC compared to healthy children. The results of the β diversity analysis revealed significant differences between the control and the FC group (Fig. 1E**).** STAMP-based analysis indicated an altered microbiota with a high abundance of *Ruminococcaceae UCG-005*, *Klebsiella*, *Ruminococcaceae NK4A214*, *Ruminiclostridium 9*, *Lachnospiraceae UCG-010*, *Ruminococcaceae UCG-004*, *Intestinimonas* and a low abundance of *Phascolarctobacterium*, *Ochrobactrum*, and *Bacillus* in the FC group compared to the control group (*P* < 0.05) (Fig. 1F).

### Serum metabolomic analysis

**Fig. 2:**
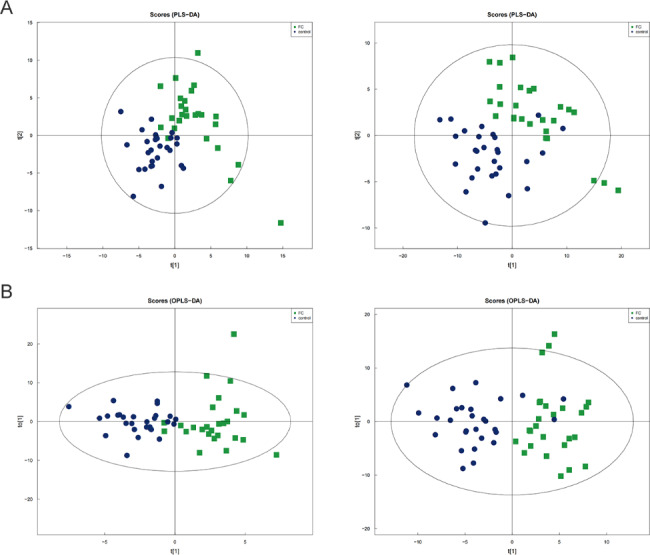
Serum metabolomic analysis. PLS-DA score plot of serum metabolites for clustering the control and functional constipation group in positive ion and negative ion, respectively **(A)**. OPLS-DA of metabolites in both ion mode from controls and functional constipation **(B)**

The tight packing of the QC samples in both positive and negative ion modes indicated that the test was repeatable (**Supplemental Fig. 2)**. As a result, changes in the test metabolic profiles may reflect biological variation between the samples. In both positive and negative ions modes, 942 metabolites were identified from healthy children and 579 metabolites were identified from constipated children. The identified metabolites mainly included lipids and lipid-like molecules, benzenoids, organic heterocyclic compounds, organic acids and derivatives (**Supplemental Fig. 3)**. The partial least squares discriminate analysis (PLS-DA), a supervised multivariate data analysis method, uncovered a clear separation between healthy controls and FC groups (Fig. 2A). Furthermore, FC group could be separated into distinct clusters clearly different from the healthy controls group in the score scatter plots of the orthogonal partial least squares discriminant analysis (OPLS-DA) model (Fig. 2B). Then, the permutation test indicated that the original OPLS-DA model was quite robust without being overfitted.

### Bioinformatics analysis of differential metabolites

**Fig. 3:**
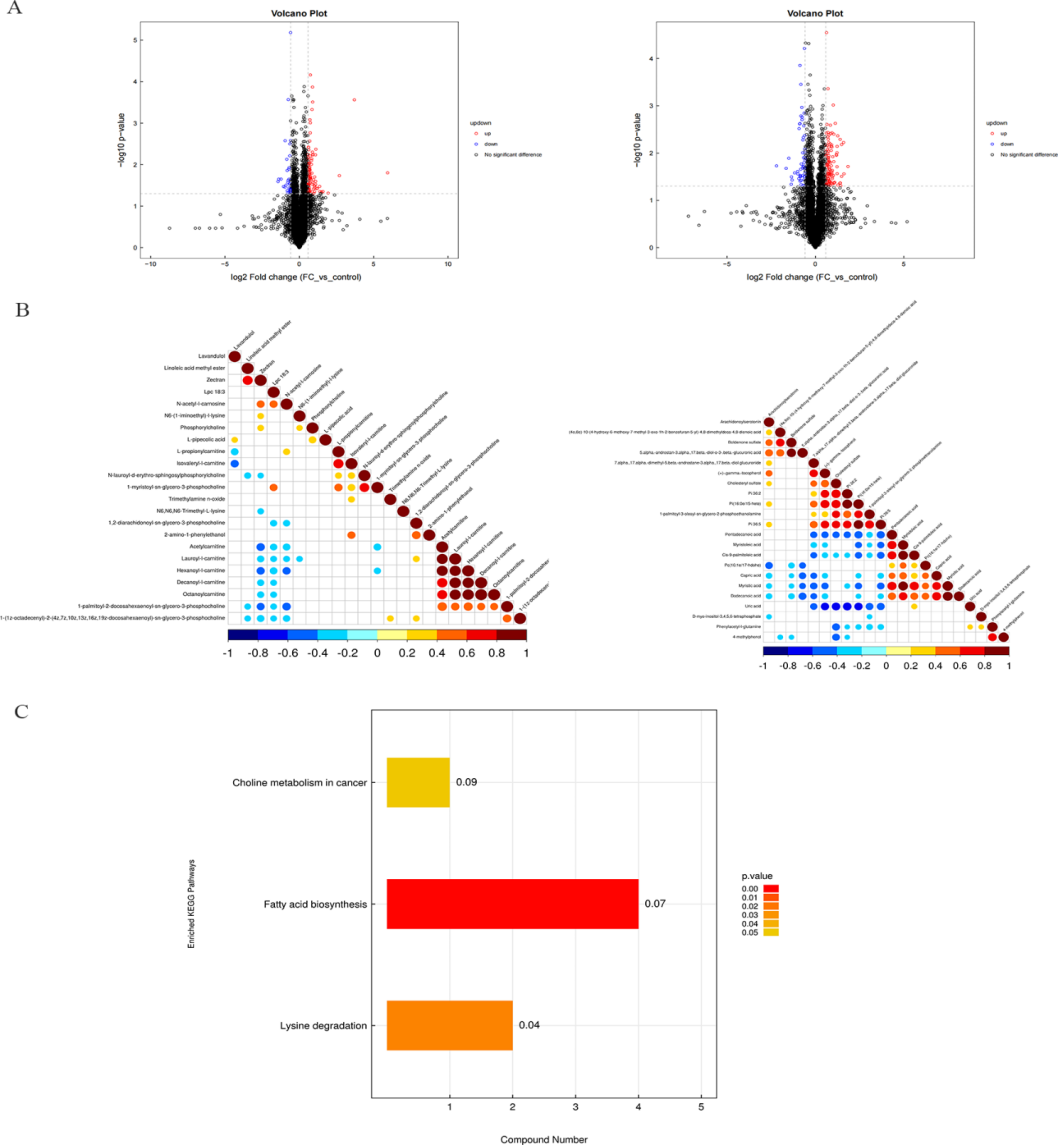
Bioinformatics analysis of differential metabolites. The univariate statistical analysis of the Volcano plot showed the metabolite changes between control and functional constipation in positive ion and negative ion mode **(A)**. The upward trend in metabolites was indicated by red scatters, the downward trend in metabolites was indicated by blue scatters, and the non-significant trend in metabolites was indicated by black scatters. The correlation heat map was used to highlight the relationship between significantly altered metabolites in positive ion and negative ion mode **(B)**. The histogram of the KEGG enrichment pathway were used to further explore the mechanism of action of gut microbiota and metabolites in children with constipation **(C)**

The univariate analysis of the volcanic map showed the significance of metabolite alterations between the FC and control groups (Fig. 3A**).** 45 different metabolites were different, including myristic acid, dodecanoic acid, phenylacetyl-l-glutamine, pentadecanoic acid, l-propionylcarnitine, and lauroyl-l-carnitine (*P* < 0.05) **(Supplemental Table 1).** The serum levels of L-PA in children with FC and healthy children were 234,435 ± 88,815 and 175,209 ± 116,993, respectively, which has significant difference between these two groups (*P* = 0.0439). The significant differences in the metabolite hierarchical clustering results are shown in **Supplemental Fig. 4**. The correlation heat map was used to visualize the significant difference in metabolites (Fig. 3B**).** KEGG pathway enrichment analysis of the expressed metabolites showed the upregulation of fatty acid biosynthesis, lysine degradation, and choline metabolism pathways (Fig. 3C). Dodecanoic acid, cis-9-palmitoleic acid, myristic acid, and capric acid were associated with the fatty acid biosynthesis pathway. Lysine degradation involved N6, N6, N6-trimethyl-l-lysine and L-PA, and choline metabolism increased phosphorylcholine circulation.

### Correlation analysis

**Fig. 4:**
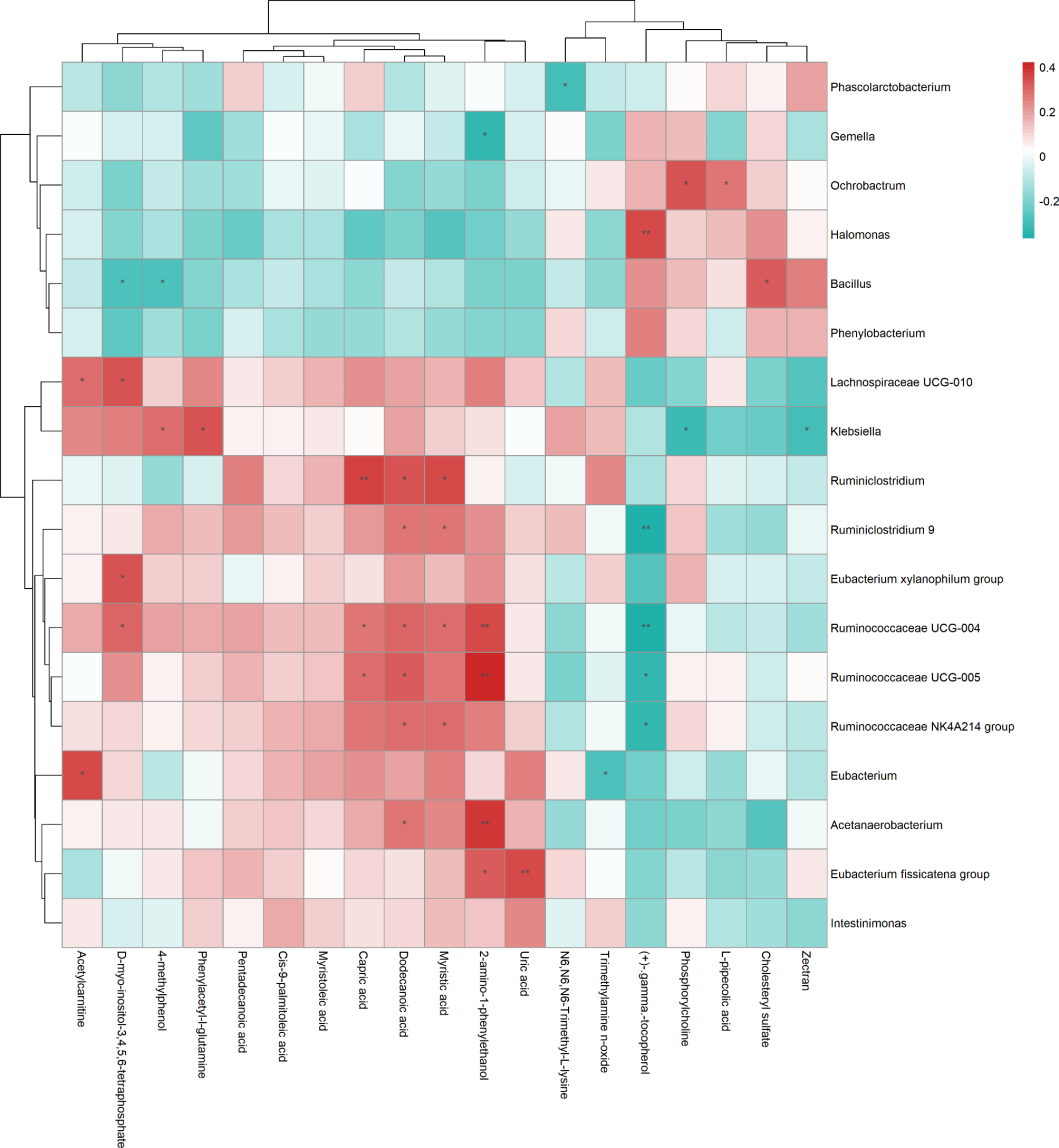
Association of differential gut microbiota with metabolites. The cluster heat map of spearman correlation hierarchical analysis of significant differences in gut microbiota and metabolites. *P < 0.05, * *P < 0.01. Pairs with low correlations (|r| < 0.5)

The correlation analysis of significant differences in gut microbiota and metabolites based on the hierarchical cluster heat map is shown in Fig. 4. For example, trimethylamine N-oxide was negatively associated with *Eubacterium*. Phosphonylcholine was positively correlated with *Ochrobactrum*. L-PA was negatively associated with *Ochrobactrum*. N6, N6, N6-trimethyl-L-lysine was positively associated with *Phascolarcrobacterium*.

### Effects of L-PA on fecal parameters and intestinal motility

**Fig. 5:**
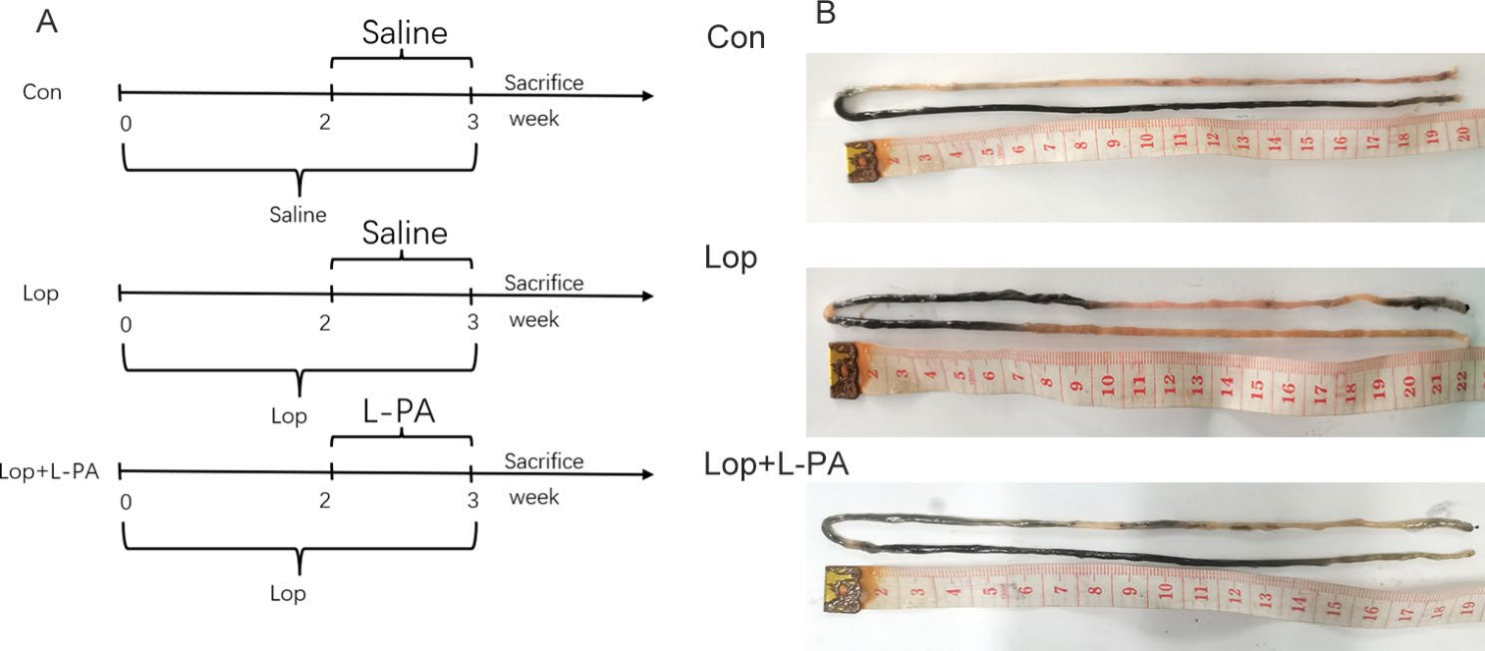
The experiment design**(A)** and small intestinal propulsion state of each group**(B)**

L-PA was administered intragastrically to mice for seven consecutive days to determine the effect of L-PA on loperamide induced constipation **(Supplemental Table 2)** [16]. The corresponding feeding chart is shown in Fig. 5A. No significant differences in the stool pallet number were noted among the three groups. However, the fecal water content was significantly reduced in the Lop group compared with the Con group (*P* = 0.0139). After administration of L-PA, the fecal water content was significantly increased (*P* = 0.0251). The results showed that the stool of constipated mice was drier than that of the control mice, and L-PA could effectively alleviate its viscosity in the constipated mice.

Compared to the Con group, the first black stool defecation time in the Lop group was significantly longer (*P* = 0.0132). After the treatment with L-PA, the first black stool defecation time of the constipated mice was significantly shortened (*P* = 0.0004). Furthermore, the intestinal transit rate of mice in the Lop group was significantly reduced compared with the Con group (*P* = 0.0022). Compared with the Lop group, the intestinal transit rate of mice in the Lop + L-PA group was increased (*P* = 0.0005). The results showed that L-PA intervention improved intestinal motility in constipated mice (Fig. 5B; Table 1**).**

**Table 1:** Effect of L-PA on excretion parameters and intestinal motility in constipated mice

	Con group	Lop group	Lop + L-PA group	*P*
Stool pellet number (ea)	7.8 ± 2.6	8 ± 4.1	10.5 ± 2.9	0.2943
Fecal water content (%)	60.5 ± 5.9	47.4 ± 4.3^**^	55.1 ± 5.7^#^	0.0025
First black stool defecation time (min)	168.5 ± 76	272 ± 38.1^*^	157 ± 37.8^##^	0.0036
Intestinal transit rate (%)	88.3 ± 9.4	69 ± 6.8^**^	91.7 ± 8.8^##^	0.0006

Data represent the mean ± SD (n = 6/group). *P < 0.05, **P < 0.01 compared with the Con group; ^#^*P* < 0.05, ^##^*P* < 0.01 compared with the Lop group.

### Levels of 5-hydroxytryptamine (5-HT) in serum and colonic tissues

**Fig. 6:**
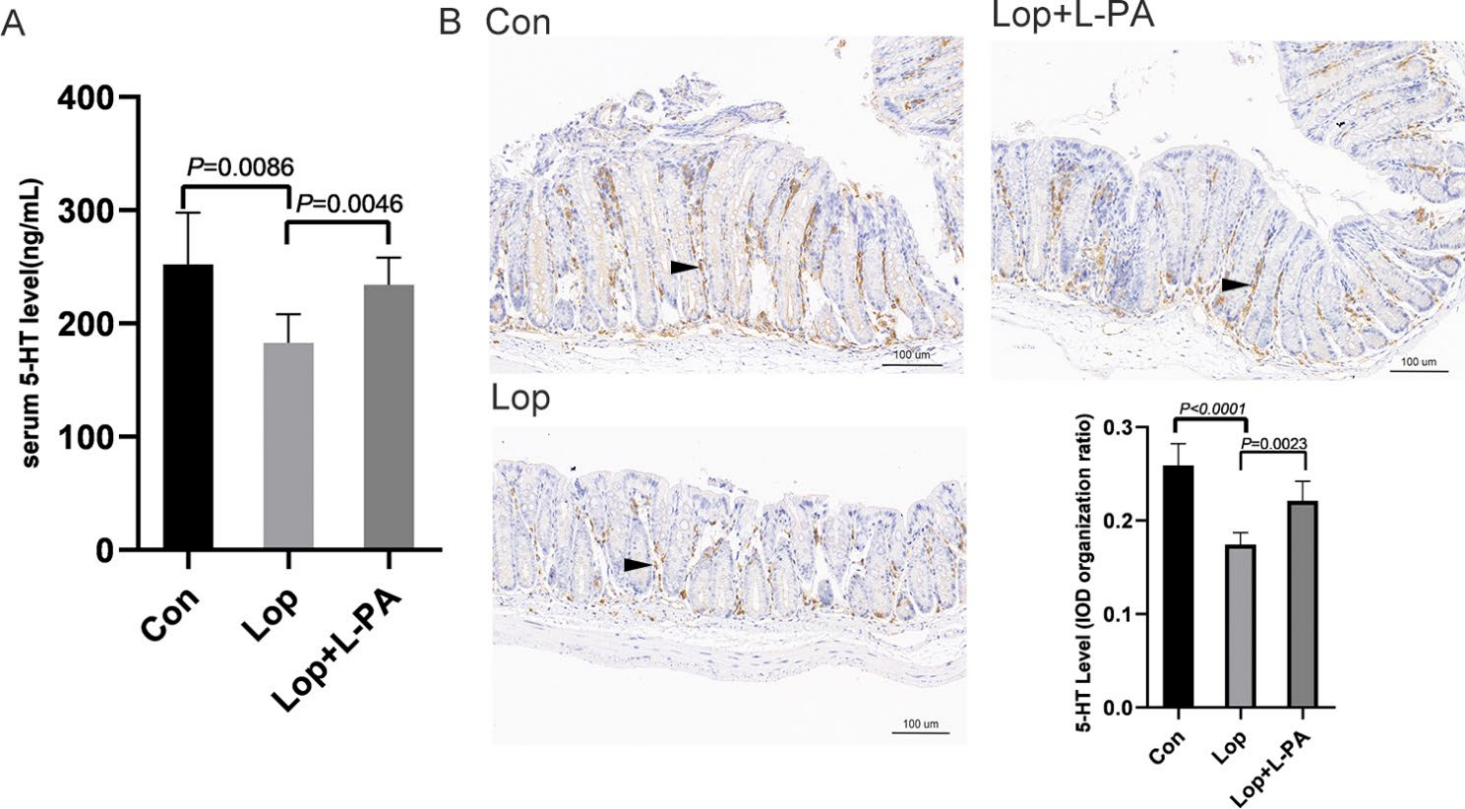
The levels of 5-HT in serum and colonic tissues. Serum levels of 5-HT in these group**(A)**. Representative immunohistochemical staining images in colonic tissues showing 5-HT (200x magnification, Bars 100 μm). Quantification using the ImageJ software for each group **(B)**. Data represent the mean ± SD (n = 6)

The serum level of 5-HT in the loperamide constipation model was significantly lower than that in the Con group (*P* = 0.0086). The serum content of 5-HT in the Lop + L-PA group was significantly increased compared to the Lop group (*P* = 0.0046) (Fig. 6A**).** In addition, immunohistochemical staining was used to examine 5-HT expression in colonic tissues (Fig. 6B**).** 5-HT was found primarily in the colonic mucosa and submucosa. The number of 5-HT-positive cells in the Lop group was significantly lower than that in the Con group (*P* < 0.0001). Nonetheless, L-PA increased 5-HT expression in constipated mice (*P* = 0.0023). These findings suggested that L-PA could influence 5-HT excretion and expression.

### Expression levels of 5-HT4R and AQP3 messenger RNA in colonic tissues

**Fig. 7:**
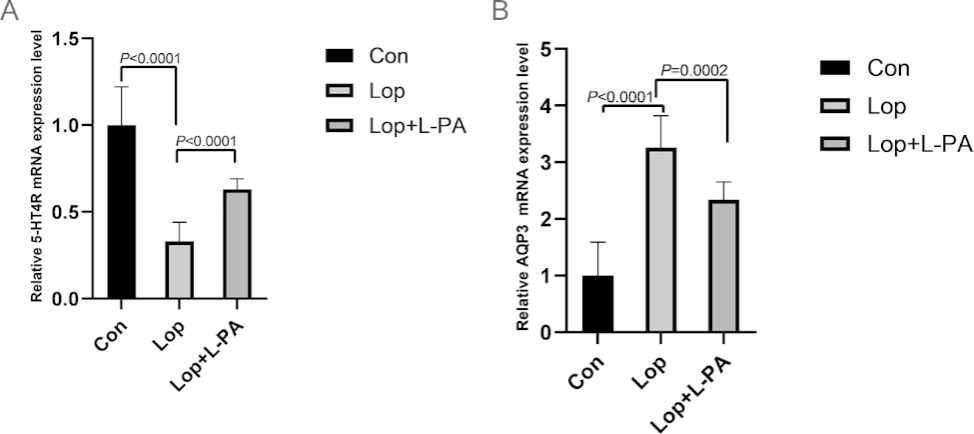
mRNA expression levels of *5-HT4R***(A)** and AQP3**(B)** in the large intestinal tissues of mice. Data represent the mean ± SD (n = 6)

The mRNA expression level of 5-HT4R in the colon tissue of the Lop group was significantly lower than that in the Con group, as shown in Fig. 7A (*P* < 0.0001). However, the expression level of 5-HT4R mRNA in the Lop + L-PA group was significantly increased compared to the Lop group (*P* < 0.0001). In contrast, the expression of AQP3 mRNA in the colon tissue of the Lop group was significantly increased compared with the Con group (*P* < 0.0001). However, after L-PA treatment, the elevated AQP3 mRNA level in the Lop group was significantly reduced (*P* = 0.0002) (Fig. 7B). The findings showed that L-PA could reverse changes in colon AQP3 and 5-HT4R mRNA levels in constipated mice.

## Discussion

FC is a common gastrointestinal disease among children and a major health problem around the globe. In recent years, gut microbiota and metabolites of microbiome have been reported to be involved in FC mechanisms. Kim et al. determined that the microbiota of FC patients had a decreased abundance of *Prevotella* and increased abundance of *Coprococcus*, *Ruminococcus*, *Blautia*, *Anaerotruncus*, and *Clostridium*[17]. In animal models, the relative abundance of *Bacteroides* and *Akkermansia* increased significantly in the fecal microbiota transplantation of the constipation group [18]. Therefore, there is evidence that FC changes the composition of the gut microbiota. In the current study, the diversity of the gut microbiota was significantly reduced in the FC group compared to the healthy group of children. The abundance of *Ruminococcaceae UCG-005*, *Klebsiella*, *Ruminococcaceae NK4A214*, *Ruminiclostridium 9*, *Lachnospiraceae UCG-010*, *Ruminococcaceae UCG-004*, and *Intestinimonas* was increased in FC, while that of *Phascolarctobacterium*, *Ochrobactrusm*, and *Bacillus* was reduced. *Phascolarctobacterium* could produce short-chain fatty acids, including acetate and propionate, and could be associated with the metabolic state and mood of the host [19]. The number of *Phascolarctobacterium faecium*-like gradually increased in younger individuals, but decreased in the elderly subjects [20]. We also found that *Phascolarctobacterium* was lowered in the fecal of children with FC. Therefore, *Phascolarctobacterium* may play a beneficial role in the human gastrointestinal tract.

Gut microbiota can modulate gut functions through the metabolites of bacterial fermentation. Attaluri et al. found that patients with slow transit constipation had higher levels of methanogenic flora compared to normal transit function constipation or control health group [21]. Butyrate concentrations were decreased significantly in mice treated with fecal microbiota transplantation from constipated patients, and after supplementation with butyrate, some constipation-related symptoms were reversed [22]. In this study, L-PA was involved in lysine degradation pathway which showed correlations with *Ochrobactrum*, which was found to be significantly lower in children with FC. These findings suggested that metabolism of the intestinal flora could influence constipation.

L-PA is a non-proteinogenic amino acid involved in the metabolic pathway that is oxidized to Δ^1^-piperideine-6-carboxylate (P6C) by the flavoenzyme pipecolate oxidase, as shown in **Supplemental Fig. 5**. Studies have showed that bacteria (*Escherichia coli*) had a vital impact on the synthesis of L-PA from L- lysine [23, 24]. The present study found that *Proteobacteria* was increased, and L-PA was reduced. Moreover, research have reported that patients with ulcerative colitis and depression/anxiety had more *Lactobacillales, Sellimonas, Streptococcus*, and *Enterococcus* but less *Prevotella_9* and *Lachnospira*, while L-PA was decreased compared to patients with ulcerative colitis without depression/anxiety [15]. Endogenous metabolites of the L-lysine derivative were altered in the colon tissue of mice suffering from slow transit constipation [25]. In this study, L-PA was found to improve fecal viscosity, reduce the first black stool defecation time and accelerate intestinal motility in Lop-induce mice in the experimental constipation animal model. The results indicated that L-PA may help with constipation.

The bacterial fermentation product, short chain fatty acids (SCFAs) and bile acid, may cause intestinal endocrine cells to release an intestinal hormone, such as 5-HT. Through various receptors on epithelial cells, smooth muscle cells, and enteric neurons, 5-HT may affect smooth muscle relaxation or contraction as well as regulate gut secretion and sensation [26, 27]. Previous research in patients with chronic constipation reported the low level expression of 5-HT in the colonic mucosa, suggesting that 5-HT might play an important role in the pathogenesis of constipation [28]. Our study showed that the expression of 5-HT in serum and colon tissue was also decreased in the Lop group compared to the Con group. However, L-PA treatment could increase the expression of 5-HT in serum and colon tissue of mice with constipation. 5-HT4R contains an important 5-HT receptor that regulates gastrointestinal function [29]. When 5-HT4R is activated, it may mediate the release of neurotransmitters critical for regulation of gastrointestinal motility by acetylcholine or substance P enteric motor neurons [30, 31]. Several studies have found that 5-HT4 receptor agonists can effectively treat constipation in both in vivo experiments and clinical trials [32, 33]. L-PA increased the expression of 5-HT4R in the colon of constipated mice in our experiment. AQP caused constipation by affecting excessive water absorption and decreasing intestinal secretions [34]. AQP3 was found to be involved in the intestinal water absorption from the luminal to the vascular side, and the overexpression of AQP3 exacerbated water absorption by the colonic mucosa, resulting in constipation [35]. In this study, L-PA was found to inhibit colon AQP3 mRNA expression in constipated mice.

## Conclusions

This study found variation in the gut microbiota and serum metabolites with a decreased level of L-PA in children with FC. L-PA treatment improved the levels of 5-HT in serum and colon tissues and reversed constipation-related indicators in loperamide induced constipated mice. L-PA upregulated the level of 5-HT4R and downregulated the expression of AQP3 in colon tissues of constipated mice. Collectively, this study suggests that L-PA might be a useful agent for the prevention or treatment of constipation.

## Methods

### Study population and sample collection

26 children with FC (14 boys and 12 girls, ranged 1–13 years old) was enrolled in this study during May 2020 to April 2021 in the inpatient department of the First Affiliated Hospital of Anhui Medical University. Patients affected by FC with infrequent bowel movements and who fulfilled the Roman IV diagnostic criteria [2]. 28 healthy children without other digestive system diseases were enrolled as control. All enrolled individuals had not taken antibiotics, probiotics and prebiotics in the past 3 months before sample collection. Blood and fecal samples were taken and the serum samples were obtained after centrifugation at 3000 r/min for 10 min. Then the serum and fecal samples were immediately stored at -80 °C until further analysis. the First Affiliated Hospital of Anhui Medical University Ethical Committee approved the study in accordance with national law and the Declaration of Helsinki. Informed consent was obtained from the parents of participants.

### 16S rDNA amplicon sequencing

The genome DNA from children’s stools was extracted using the cetyltrimethylammonium bromide (CTAB) techniques, and then the purity and concentration of the DNA were determined through agarose gel electrophoresis. Sequencing regions of chosen V3-V4 variable regions were amplified using high-fidelity DNA polymerase and specific primers. The PCR products were collected using 2% agarose gel electrophoresis to determine and collect the desired segment. PCR recovery products were quantified using QuantiFluor-ST™ Blue Fluorescence Quantification System (Promega) and blended using equidensity ratios while purifying the mixture with AxyPrepDNA Gel recovery kit (AXYGEN). The library was quality checked using the Agilent Bioanalyzer 2100 and the Qubit. It was built using the NEB Next® UltraDNA™Library Prep Kit. Following quality control, the library was subjected to hands-on sequencing.

### Data processing and statistical analysis

After trimming the Barcode and primer sequences, paired-end reads were merged using FLASH software. The raw tags were performed under require strict filtering to obtain clean reads. Uparse was used to cluster all the clean reads of all samples. The sequences were clustered into OTUs with ≥ 97% similarity. The taxonomy information was annotated using the Ribosomal Database Project classifier. Taxonomy-based analyses were conducted by classifying each sequence using the SILVA database.

Simultaneously, OTUs were calculated to estimate α diversity, and reveal changes in species diversity and richness between the groups. The Venn diagram was used to display information about common and distinct OTUs in different groups. Furthermore, OTUs were compared to multiple sequences to calculate phylogenetic distance in order to learn more about community structure. The QIIME 1.9.1 software package was then used to calculate β diversity. The STAMP 2.1.3 was used to validate alteration in the individual species richness within different groups. T-test and Wilcoxon test were used to test the significance of the grouped samples, and *P* < 0.05 was considered statistically significant.

### Sample preparation and Metabolomics analysis

After thawing the sample at 4 °C, 100 µL sample and 400 µL cold methanol/acetonitrile solution (1:1, v/v) were mixed. Then, the mixture was centrifuged at speed of 14,000 g for 4 min at 4 °C, the supernatant was dried in vacuum and then resolved in 100 µL aqueous acetonitrile (acetonitrile: water = 1:1, v/v), vortexed, and centrifuged at 14,000 g for 5 min at 4 °C to collect the supernatant for analysis.

To separate the samples, an Ultra High Performance Liquid Chromatography System (1290 Infinity LC, Agilent Technologies) was used. The mobile phase in both ESI positive and negative modes contained A (25 mM ammonium acetate and 25 mM ammonium hydroxide in water) and B (acetonitrile). The gradient had a constant temperature of 25 °C and a flow rate of 0.3 mL/min with 2uL injection volume. It comprised of: 0-1 min, B 85%; 1–12 min, B 85%-65%; 12-12.1 min, B 65% − 40%; 12.1–15 min, B remained at 40%; 15-15.1 min, B 40 − 85%; 15.1–20 min, B maintained at 85%.

For mass spectrometry analysis, a Triple TOF 6600 Mass Spectrometer (AB SCIEX) was used. The ESI source conditions were as follows: Ion Source Gas1 and Gas2: 60, Curtain gas: 30, source temperature: 600 °C, Ion spray voltage floating ± 5500 V; TOF MS scan m/z range and accumulation time: 60-1000 Da and 0.20 s/spectra, product ion scan m/z range and accumulation time: 25-1000 Da and 0.05 s/spectra. The product ion scan was obtained using information dependent acquisition (IDA) and high sensitivity mode.

### Data processing and statistical analysis

The data were then subjected to peak extraction and metabolite identification using XCMS. The normalized data were used in multivariable analysis with R 2.15.3 and SIMCA-P 16.1 software that included partial least squares discriminant analysis (PLS-DA) and orthogonal partial least squares discriminant analysis (OPLS- DA). The model robustness was evaluated using the response permutation test. In the OPLS-DA model, compute the variable importance for the projection value (VIP) to demonstrate its impact on categorization. The student’s t-test and fold change analysis were used to calculate significant differences between groups, and then significantly altered metabolites with VIP > 1 and *P* < 0.05 were screened. Pearson’s correlation analysis was used to determine the correlation between the two variables. Fisher’s exact test was used to KEGG pathway enrichment analysis, *P* < 0.05 was considered as statistically significant.

### Correlation analysis

The relative abundance of the 18 significant microbiota in genus levels (*P* < 0.05) and 45 significant differential metabolites (VIP > 1, *P* < 0.05) were organized in a table as an input file for subsequent analysis. The spearman correlation analysis was used to analyze the correlation coefficient between the significantly different microbiota and the significant differential metabolites, and the hierarchical cluster heat map was carried out in combination with R 2.15.3 software.

### Animal experiment

The Ethical Committee of Anhui Medical University approved the study protocols, which were carried out in accordance with the ARRIVE guidelines and the guidelines for the care and use of laboratory animals (Directive 1,182,010/63/EU). Female mice (C57BL/6) at 6–8 weeks of age weighing 20 g ± 2 g were purchased from Henan Sikebas Biotechnology Co., Ltd. All animals were kept in the Experimental Animal Center of Anhui Medical University. They were fed freely in standard animal cage conditions and were allowed to adapt to the laboratory conditions for one week before the experiment. They were then randomly divided into three groups (n = 6/ group). These groups were the control group (Con), the constipation model group (Lop), and the L-PA treatment group (Lop + L-PA). The Lop and Lop + L-PA groups were given loperamide (9.8 mg/kg of body weight, in saline) by intragastric administration twice a day for 14 days, while mice in the Con group were given only saline. Following successful modeling, mice in the Lop + L-PA group were given L-PA (250 mg/kg dissolved in saline, once a day) and loperamide by intragastric administration; the Lop group was given loperamide and saline, while the control group was given normal saline for one week.

### Measurement of constipation parameters

Indian ink (Yuanye, Shanghai, China) was gavaged into the stomach and timed to thirty mins after the last dose of medication. Each mouse was placed in its own cage with free access to water, and the time of the first black defecation was recorded. Within 6 h, the number and weight of feces excreted by the mice were also counted. Fecal samples were collected and dried immediately after observation. The water content of feces was determined using the following formula:

Fecal water content (%) = [fecal wet weight (g)-fecal dry weight (g)]/ fecal wet weight (g) × 100.

### Intestinal transit test

All mice were fasted for 12 h before being given water. The mice were sacrificed after being gavaged with Indian ink for 25 min, and the small intestine was quickly obtained. The entire length of the small intestine and the distance traveled by Indian ink were measured. The following formula was used to calculate the intestinal transit rate:

Intestinal transit rate (%) = [The distance traveled by Indian ink (cm) / Whole length of small intestine (cm)] × 100.

### Determination of serum 5-hydroxytryptamine (5-HT) level in mice

Blood samples were collected following experiment. After 2 h at room temperature, serum was obtained by centrifugation at 3000 rpm for 10 min at 4 °C. All serum samples were collected separately and stored in -80 °C freezer. The content of 5-HT was evaluated using manufacturer instructions of the ELISA kit (ColorfulGene, Wuhan, China).

### Immunohistochemistry

The expression level of 5-HT was detected by immunohistochemistry. The colon paraffin sections were routinely fixed with 4% paraformaldehyde for 10 min. After three phosphate buffer rinses (3 min each), peroxidase blocking solution was added dropwise and slides were incubated for 10 min at room temperature. The slides were then incubated overnight with anti-5-HT antibodies (Abcam, Cambridge, UK) at 4 °C, followed by HRP-conjugated anti-rabbit IgG at room temperature for 30 min and stained with diaminobenzidine. The cells that were colored were brownish yellow. For analysis, ImageJ 6 software was used. The expression of 5-HT per unit area of colonic tissue was randomly observed using a high-power microscope (x200). The optical density of 5-HT expression in mice from each group was statistically measured.

### Quantitative real-time polymerase chain reaction (qRT-PCR)

The expression level of 5-HTR4 and AQP3 was measured using qRT-PCR. Total RNA was extracted from colon tissues using Trizol reagent (Life Technologies, USA), and then transcribed to the complement DNA (cDNA) using PrimeScript™RTreagent Kit with gDNA Eraser (TaKaRa, Beijing, China). Amplification was performed from 10 µL reaction (including 5 µL of 2×SYBR Green mixture, 1 µL of cDNA template, 1 µL of forward primer, 1µL of reverse primer, and 2 µ L of RNase free water). The reaction condition was as follows: initiation at 95 °C, 10 min, 40 cycles of 95 °C for 20 s and 60 °C, for 1 min. β-actin was used as reference gene to calculate the relative expressions of target genes. The forward and reverse primers for the genes are shown in **Supplemental Table 3**.

### Statistical analysis for animal experiment

The data was analyzed using SPSS software. The experiment data were expressed as mean ± standard deviation. The independent-samples t-test was used to analyze the comparison between the two groups. The one-way analysis of variance (ANOVA) was used for multiple groups, and *P* < 0.05 was considered statistically significant.

## Electronic supplementary material

Below is the link to the electronic supplementary material.

Supplementary Material 1

Supplementary Material 2

Supplementary Material 3

Supplementary Material 4

Supplementary Material 5

Supplementary Material 6

Supplementary Material 7

Supplementary Material 8

## Data Availability

The datasets generated and/or analysed during the current study are available in NCBI’s Sequence Read Archive (SRA) repository under the BioProject ID PRJNA839724. (https://www.ncbi.nlm.nih.gov/sra/PRJNA839724)

## Abbreviations

FC: Functional constipation
UPLC-Q/TOF-MS: Ultra-performance liquid chromatography/quadrupole time of flight-mass spectrometry
Lop: Loperamide
OTUs: Operational taxonomic units
PLS-DA: Partial least squares discriminate analysis
OPLS-DA: Orthogonal partial least-squares discriminant analysis
VIP: Variable importance for the projection
L-PA: L-pipecolic acid
CTAB: Cetyltrimethylammonium bromide
IDA: Information dependent acquisition
5-HT: 5-hydroxytryptamine
qRT-PCR: Quantitative real-time polymerase chain reaction
