# Supplementary material for: Protective effect of L-pipecolic acid on constipation in C57BL/6 mice based on gut microbiome and serum metabolomic
Source: BMC Microbiol. 2023 May 20;23:144. doi: 10.1186/s12866-023-02880-3 (PMC10199545; doi:10.1186/s12866-023-02880-3)
Supplement: Supplementary file 2 — Supplementary Material 2 [file 12866_2023_2880_MOESM2_ESM.pptx]

## Slide 1
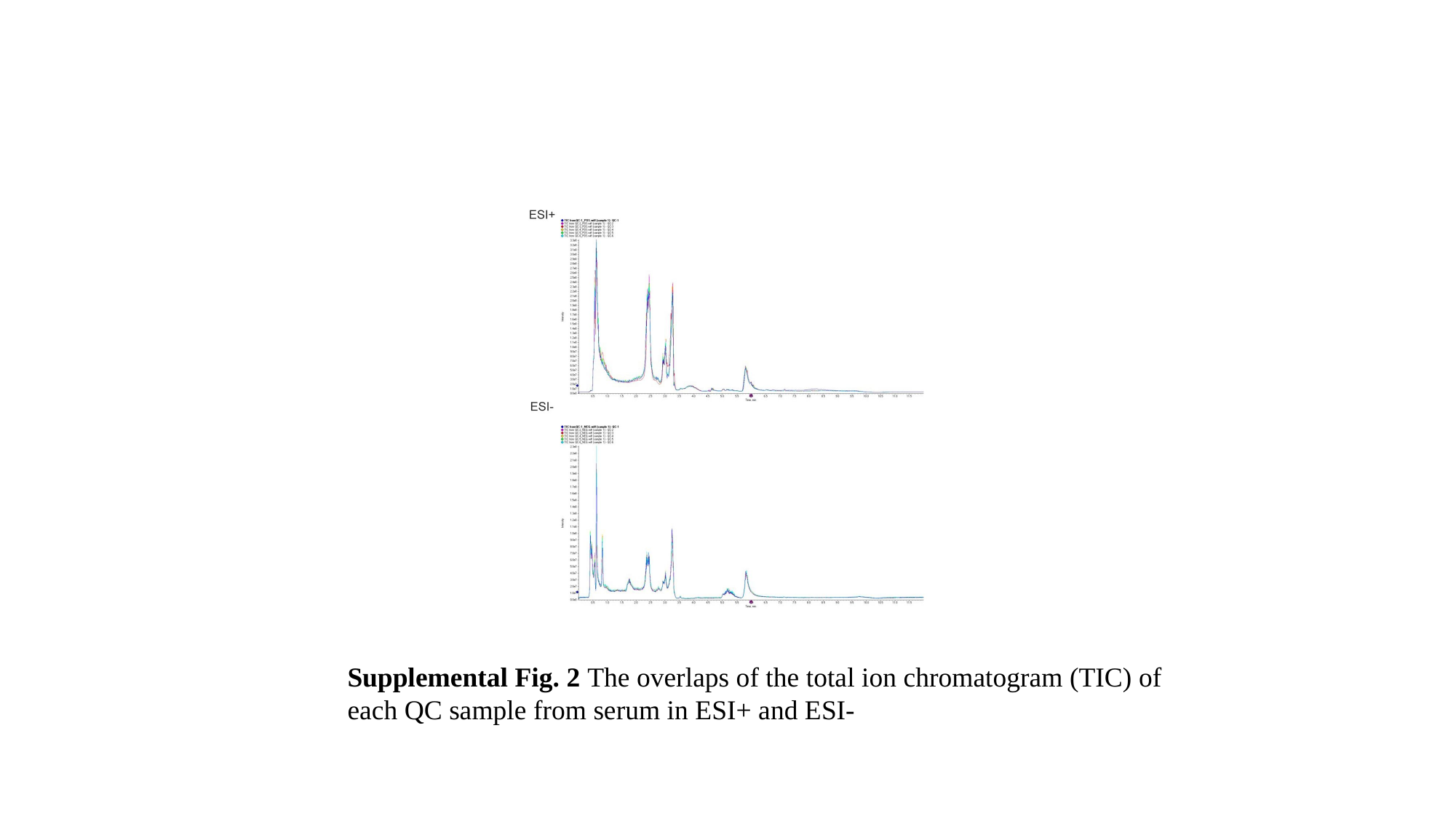

Supplemental Fig. 2 The overlaps of the total ion chromatogram (TIC) of each QC sample from serum in ESI+ and ESI-
