# Supplementary figures and images for: Protective effect of L-pipecolic acid on constipation in C57BL/6 mice based on gut microbiome and serum metabolomic
Source: BMC Microbiol. 2023 May 20;23:144. doi: 10.1186/s12866-023-02880-3 (PMC10199545; doi:10.1186/s12866-023-02880-3)

## Slide 1
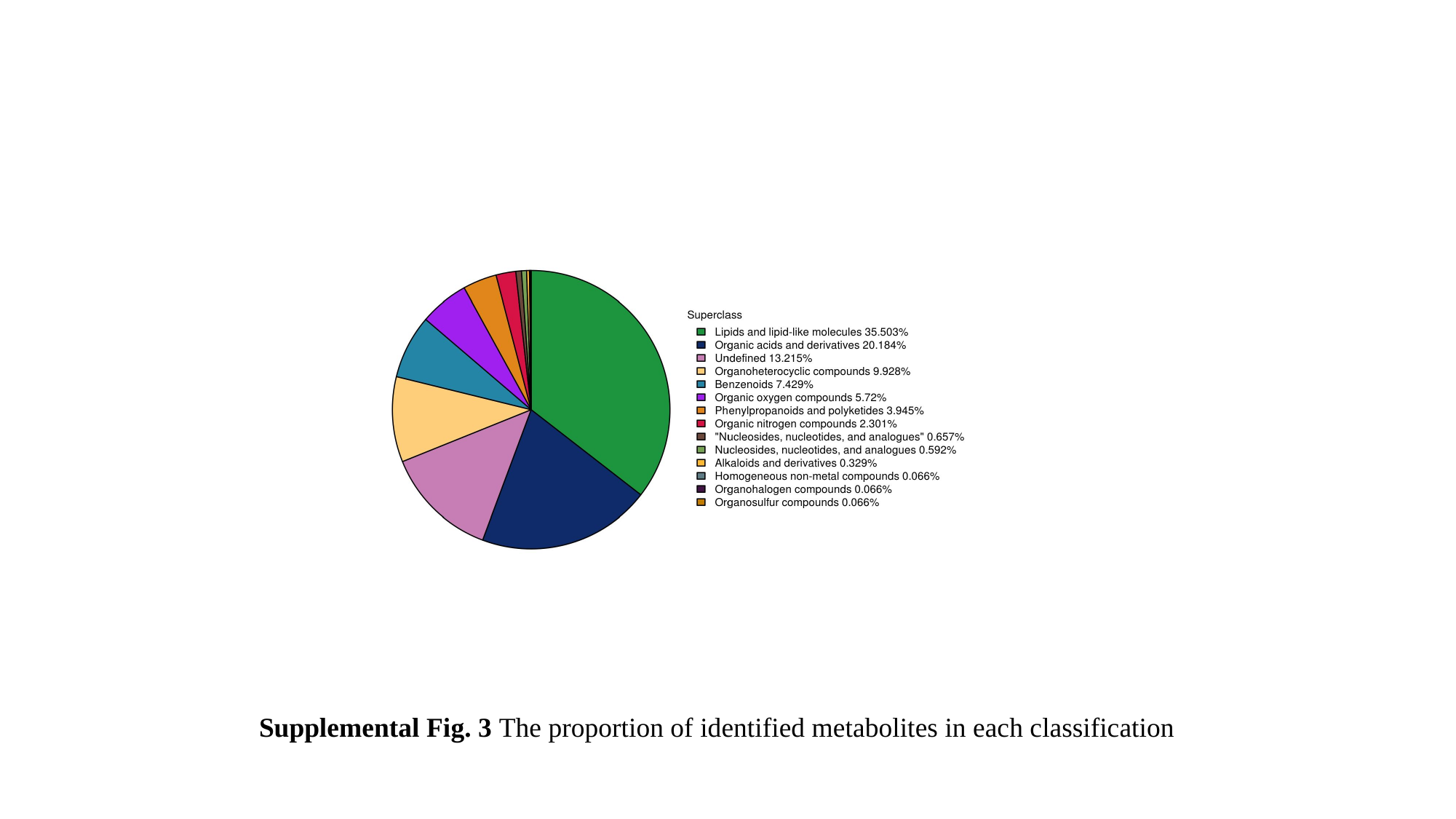

Supplemental Fig. 3 The proportion of identified metabolites in each classification

Supplement: Supplementary file 3 — Supplementary Material 3 [file 12866_2023_2880_MOESM3_ESM.pptx]
