# Supplementary material for: Protective effect of L-pipecolic acid on constipation in C57BL/6 mice based on gut microbiome and serum metabolomic
Source: BMC Microbiol. 2023 May 20;23:144. doi: 10.1186/s12866-023-02880-3 (PMC10199545; doi:10.1186/s12866-023-02880-3)
Supplement: Supplementary file 6 — Supplementary Material 6 [file 12866_2023_2880_MOESM6_ESM.pdf]

Supplemental Table. 1 Differential metabolites

	Name	VIP	Fold change	p-value	m/z	rt(s)
[M-H]-	7.alpha.,17.alpha.-dimethyl-5.beta.-androstane-3.alpha.,17.beta.-diol glucuronide	2.291293	0.546143	0.00014	495.3114	23.875
[M-H]-	Phenylacetyl-l-glutamine	2.916856	2.052622	0.003969	263.1025	228.9655
[M-H]-	Myristic acid	5.889783	1.698879	0.004157	227.2005	50.8055
(M-H)-	Dodecanoic acid	6.063163	3.015407	0.005968	199.1693	52.6645
[M-H]-	Pentadecanoic acid	2.124153	1.594902	0.006948	241.2157	50.137
[M-H]-	Pi 36:2	5.575839	0.74034	0.008508	861.5454	198.467
[M-H]-	D-myo-inositol-3,4,5,6-tetraphosphate	1.670227	2.285193	0.011103	498.9261	22.8875
[M-H]-	Pi(16:0e/15-hete)	4.79455	0.736883	0.012746	859.5279	197.5825
[M-H]-	Capric acid	2.16562	1.726647	0.01984	171.1382	55.376
[M+Hac-H]-	Pc(16:1e/17-hdohe)	1.731669	1.221294	0.019865	864.5703	142.144
[M-H]-	1-palmitoyl-3-oleoyl-sn-glycero-2-phosphoethanolamine	1.885809	0.722712	0.021926	716.5162	39.56
[M-H]-	Cholesteryl sulfate	9.679171	0.720623	0.022106	465.3016	25.8965
[M-H]-	4-methylphenol	8.438977	1.674644	0.026765	107.0498	25.691
[M-H]-	Boldenone sulfate	1.138137	0.551519	0.030844	365.139	29.726
(M-H)-	Myristoleic acid	2.312453	1.500492	0.031578	225.1844	51.3635
[M-H]-	(4e,8e)-10-(4-hydroxy-6-methoxy-7-methyl-3-oxo-1h-2-benzofuran-5-yl)-4,8-dimethyldeca-4,8-dienoic acid	1.988573	0.391386	0.031896	387.1805	54.1125
[M-H]-	(+)-.gamma.-tocopherol	1.194959	0.722424	0.032725	415.3537	32.2915
[M-H]-	Cis-9-palmitoleic acid	5.606776	1.36567	0.037742	253.2159	48.8415
[M-H]-	Pi 36:5	1.206994	0.570276	0.037918	855.4962	197.168
[M-H]-	Uric acid	1.149823	1.716052	0.038669	167.02	333.0345
[M-H-H2O]-	5.alpha.-androstan-3.alpha.,17.beta.-diol-o-3-.beta.-glucuronic acid	2.886325	0.388032	0.045593	449.251	189.486
[M-H]-	Arachidonoylserotonin	1.456546	0.649635	0.048547	461.3214	185.706
[M+H]+	1-palmitoyl-2-docosahexaenoyl-sn-glycero-3-phosphocholine	18.9003	1.23245	0.004202	806.5686	142.629
[M+H]+	L-propionylcarnitine	3.091645	1.329844	0.006218	218.1377	286.2215
[M+H]+	Lauroyl-l-carnitine	3.144338	1.604004	0.011437	344.2777	184.8035

[M+H-CH4O]+	Linoleic acid methyl ester	1.829013	0.715118	0.013909	263.2357	107.564
[M+H]+	Trimethylamine n-oxide	1.790077	1.790517	0.016024	76.07519	337.4685
[M+H]+	1,2-diarachidonoyl-sn-glycero-3-phosphocholine	1.214832	1.530803	0.018229	830.5504	160.8165
[M+H]+	Zectran	2.628169	0.726192	0.02123	223.168	122.8105
[M+H]+	N-lauroyl-d-erythro-sphingosylphosphorylcholine	1.522277	1.701434	0.023198	647.5087	185.359
[M+H]+	Lpc 18:3	4.490182	0.807361	0.024939	518.3201	196.894
[M+H]+	1-(1z-octadecenyl)-2-(4z,7z,10z,13z,16z,19z-docosahexaenoyl)-sn-glycero-3-phosphocholine	3.30032	1.159447	0.025277	818.6028	139.3895
[M+H]+	Phosphorylcholine	6.117702	0.83671	0.028224	184.0722	142.276
[M+H]+	Isovaleryl-l-carnitine	1.616931	1.257962	0.030868	246.1689	244.5185
[M+H]+	Hexanoyl-l-carnitine	2.377498	1.526111	0.032025	260.1844	226.811
[M+H]+	N6,N6,N6-Trimethyl-L-lysine	1.243355	1.527203	0.032527	189.1583	536.4
[M+H]+	Decanoyl-l-carnitine	4.447512	1.485847	0.033406	316.2467	193.109
[M+H]+	Octanoylcarnitine	3.781487	1.487192	0.034366	288.2168	205.658
(2M+H)+	Lavandulol	8.285491	0.734678	0.037021	309.2776	53.421
[M+H]+	N-acetyl-l-carnosine	1.00606	0.754853	0.038529	269.1232	338.6995
[M+H]+	N6-(1-iminoethyl)-l-lysine	2.617681	0.88684	0.040928	188.1385	528.131
[M+H]+	1-myristoyl-sn-glycero-3-phosphocholine	2.278657	1.492011	0.042806	468.3068	200.0475
[M+H]+	L-pipecolic acid	2.486865	0.747368	0.043939	130.0853	545.525
[M+H-H2O]+	2-amino-1-phenylethanol	2.769685	1.158774	0.047558	120.0801	269.6865
[M+H]+	Acetylcarnitine	18.58906	1.30082	0.049703	204.1228	312.532

VIP > 1 and p-value < 0.05 were used to screen significantly changed metabolites in positive ion and negative ion
