# Supplementary material for: Protective effect of L-pipecolic acid on constipation in C57BL/6 mice based on gut microbiome and serum metabolomic
Source: BMC Microbiol. 2023 May 20;23:144. doi: 10.1186/s12866-023-02880-3 (PMC10199545; doi:10.1186/s12866-023-02880-3)
Supplement: Supplementary file 7 — Supplementary Material 7 [file 12866_2023_2880_MOESM7_ESM.pdf]

Supplemental Table. 2 Constipation model preparation

	Con group	Lop group	Lop+L-PA group
Stool pellet number of 6h(ea)	8.3±2.7	3.7±2.1*	2.8±3.6*
First black stool defecation time (min)	191.8±8.3	330.7±54.0**	366.7±79.4**

Data represent the mean ± SD. *$P < 0.05$, **$P < 0.01$ compared with Con group
