# Supplementary material for: Protective effect of L-pipecolic acid on constipation in C57BL/6 mice based on gut microbiome and serum metabolomic
Source: BMC Microbiol. 2023 May 20;23:144. doi: 10.1186/s12866-023-02880-3 (PMC10199545; doi:10.1186/s12866-023-02880-3)
Supplement: Supplementary file 8 — Supplementary Material 8 [file 12866_2023_2880_MOESM8_ESM.pdf]

Supplemental Table. 3 Sequences of Primers Used in this study

Gene	Amplicon Size （bp）	Forward primer （5'→3'）	Reverse primer （5'→3'）
β-actin	120	AGTGTGACGTTGACATCCGT	TGCTAGGAGCCAGAGCAGTA
5-HT4R	122	GTTCGTGTCAAAACTGATCC	AATCAAAGTGGACTGATCCC
AQP3	145	AAATGGACTTCTAGGCTTGG	AAACTTACGACCTGGACTTC
